# An extensive assessment of the impacts of BaO on the mechanical and gamma-ray attenuation properties of lead borosilicate glass

**DOI:** 10.1038/s41598-024-56040-2

**Published:** 2024-03-05

**Authors:** M. I. Sayyed, K. A. Mahmoud, Jack Arayro, Yasser Maghrbi, M. H. A. Mhareb

**Affiliations:** 1 Department of Physics, Faculty of Science, Isra University, Amman, Jordan; 2 Renewable Energy and Environmental Technology Center, University of Tabuk, 47913 Tabuk, Saudi Arabia; 3 Nuclear Materials Authority, P.O. Box 530, El-Maadi, Cairo, Egypt; 4 Ural Federal University, 19 Mira St., Yekaterinburg, Russia 620002; 5 College of Engineering and Technology, American University of the Middle East, 54200 Egaila, Kuwait; 6 University of Tunis El Manar, 2092 Tunis, Tunisia; 7 Université Côte d’Azur, 06100 Nice, France; 8 Department of Physics, College of Science, Imam Abdulrahman Bin Faisal University, Box 1982, 31441 Dammam, Saudi Arabia; 9 Basic and Applied Scientific Research Center, Imam Abdulrahman Bin Faisal University, P.O. Box 1982, 31441 Dammam, Saudi Arabia

**Keywords:** Borosilicate glasses, Barium oxide, Radiation shielding, Mechanical properties, Materials science, Physics

## Abstract

The current work deals with the synthesis of a new glass series with a chemical formula of 5Al_2_O_3_–25PbO–10SiO_2_–(60-x) B_2_O_3_–xBaO; x was represented as 5, 10, 15, and 20 mol%. The FT-IR spectroscopy was used to present the structural modification by rising the BaO concentration within the synthesized glasses. Furthermore, the impacts of BaO substitution for B_2_O_3_ on the fabricated borosilicate glasses were investigated using the Makishima-Mackenzie model. Besides, the role of BaO in enhancing the gamma-ray shielding properties of the fabricated boro-silicate glasses was examined utilizing the Monte Carlo simulation. The mechanical properties evaluation depicts a reduction in the mechanical moduli (Young, bulk, shear, and longitudinal) by the rising of the Ba/B ratio in the fabricated glasses. Simultaneously, the micro-hardness boro-silicate glasses was reduced from 4.49 to 4.12 GPa by increasing the Ba^2+^/B^3+^ ratio from 0.58 to 3.18, respectively. In contrast, the increase in the Ba/B ratio increases the linear attenuation coefficient, where it is enhanced between 0.409 and 0.448 cm^−1^ by rising the Ba^2+^/B^3+^ ratio from 0.58 to 3.18, respectively. The enhancement in linear attenuation coefficient decreases the half-value thickness from 1.69 to 1.55 cm and the equivalent thickness of lead is also reduced from 3.04 to 2.78 cm, at a gamma-ray energy of 0.662 MeV. The study shows that the increase in the Ba^2+^/B^3+^ ratio enhances the radiation shielding capacity of the fabricated glasses however, it slightly degrades the mechanical properties of the fabricated glasses. Therefore, glasses with high ratios of Ba^2+^/B^3+^ have high gamma-ray shielding ability to be used in hospitals as a shielding material.

## Introduction

Radioactive materials are being extensively used in technologies of this century. Different medical, agricultural, and space research sectors are now relying on these materials. Depending on their application, various levels of radiation are being exploited; the amounts of energy emitted by these sources can be harmful to humankind. Acute exposure to high energy levels of ionizing radiation can cause major health issues, damaging the body cells, and leading in some cases to genetic disorders^1–5^. It is then crucial to manipulate radioactive materials with a high level of caution and to protect the working personnel from any radiation hazard. Therefore, shielding materials are being used as a barrier to these offensive radiations^6–9^. Research on radiation shielding and radiation attenuation has revealed a wide range of materials that are of high efficiency in reducing radiation levels^10–13^. The two classic materials commonly adopted as shielding materials are concrete and lead. Although they possess numerous advantages, these classical shielding materials suffer from many drawbacks. For instance, concrete is heavy and bulky, causing challenges in its transportation and installation^14,15^. Moreover, concrete can degrade over time when exposed to moisture, affecting its integrity, and thus endangering its shielding performance. On the other hand, lead-based shielding materials are highly toxic, they are poor in flexibility and chemical stability. Therefore, developing novel shielding materials, that hold great shielding properties, is highly necessary to replace conventional ones^16^.

Extensive research has been done on alternative radiation shielding materials, suggesting glass as an effective solution^17–19^. In fact, glass is highly abundant and of competitive price, resulting in being very accessible. Additionally, glass is easy to process and does not require extensive maintenance. Most importantly, glass is optically transparent, which makes its employment very convenient for numerous shielding applications^20–22^. The merge of different oxides in the glass system gives it diversity in characterization and enhancement in density, effectively improving the absorption of gamma rays.

Different types of glasses, such as silicate, borate, tellurite, phosphate, and antimonate, are available, but a few glasses were used in different applications due to their low structural stability. Soda-lime glass is a famous glass type widely used in different applications. Still, it has many drawbacks, such as limited thermal shock resistance, susceptibility to chemical attack, and relatively low mechanical strength^23^. At the same time, borate glass has lower chemical durability and a higher coefficient of thermal expansion, which leads to limited use. The mixing of borate and silicate gives a mixture of two glasses called borosilicate, which has desirable properties such as lower thermal expansion and high durability^24^. This type of glass in a radiation shielding field is a good idea due to its properties. The addition of oxides to the glass system changes the glass structure and enhance the mechanical and optical properties of the glass. Heavy oxides such as PbO and BaO can increase the glass density and improve the shielding properties, while Al_2_O_3_ can play two roles: glass modifier or glass former, according to the ratio in the glass system^25,26^.

In particular, it has been proven that borosilicate glasses, adopted in the vitrification of nuclear waste, are highly efficient in stabilizing the High-Level Liquid Waste through the suppression of its migration into the natural environment^27,28^. For example, borosilicate glass is known to be water and chemical-resistant, it possesses a lower melting point than classic silicate glass, which increases its lifetime^29^. Moreover, concerning their applications in radiation protection, oxide incorporation is found to improve the radiation-shielding abilities for glass materials in absorbing various types of ionizing radiation^30^.

Many studies have explored borosilicate glass, especially in the radiation shielding field^31–35^. Cheewasukhanont et al.^31^ studied the impact of particle size on radiation shielding properties for the (55-x)SiO_2_–xBi_2_O_3_–20B_2_O_3_–10CaO–15Na_2_O glass system where x = 0, 5, 10, 15, 20 and 25 mol%. The particle size did not affect glass density, while nano-sized particles showed better-shielding properties than microsized particles. Chanthima and Kaewkhao^32^ investigated the radiation shielding for 23Na_2_O–15B_2_O_3_–2Al_2_O_3_–10CaO–(50-x)SiO_2_–xBi_2_O_3_ where x = 0, 5, 10, 15, and 20 mol%. Adding Bi_2_O_3_ instead of SiO_2_ increased the radiation shielding ability of the glass system. Mhareb^35^ explored the structural, mechanical, optical, and radiation shielding properties for 10SiO_2_–10TiO_2_–30SrO–(49.5-x)B_2_O_3_–xGd_2_O_3_ glass and glass ceramics where x = 0.5, 1, 1.5, and 2 mol%. Adding Gd_2_O_3_ led to a slight variation in mechanical and optical properties. At the same time, the shielding properties showed gradual enhancement.

The current work is innovative in that it examines the effects of increasing the Ba^2+^/B^3+^ ratio on the mechanical, structural, and gamma-ray shielding characteristics of a newly synthesized boro-silicate glass consisting of Al_2_O_3_-PbO-SiO_2_-B_2_O_3_-BaO.

## Materials and methods

### Glasses preparation

**Figure 1:**
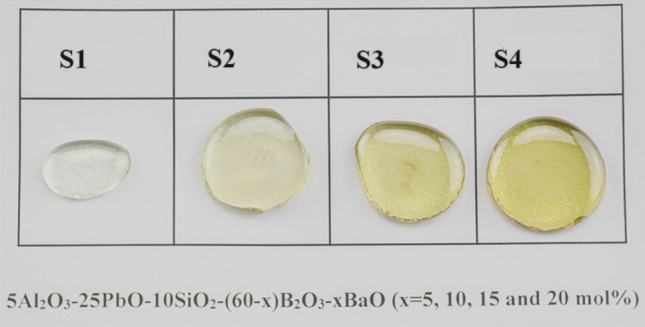
A picture of the synthetic glasses demonstrated how the color changed as the Ba/B ratio increased.

To prepare the investigated glasses, of general formula 5Al_2_O_3_–25PbO–10SiO_2_–(60-x) B_2_O_3_–xBaO, where x was varied between 15, 10, 15, and 20 mol%, the melt quenching method was employed. The following oxides: MgO (purity of 99.99%), BaO (purity of 99.99%), SiO_2_ (purity of 99.99%), B_2_O_3_ (purity of 99.99%), and PbO (purity of 99.99%) were supplied by Sigma Aldrich (USA) and used for fabrication of the glasses in the current work. The required amounts of each metal oxide were accurately weighed and mixed using agate mortar to ensure a homogeneous and uniform glass structure. The powders in high-purity alumina crucibles were put in an electric furnace at 1100 °C for two hours. The glass was carefully poured into a metal mold once it had totally liquefied. The glasses followed an annealing procedure in a different furnace, where they were heated to 400 °C for 4 h, in order to reduce their internal stress. Figure 1 represents a photo of the prepared glass samples. In the mentioned figure the Ba/B ratios are 0.58 (for S1 glass sample), 1.27 (for S2 glass sample), 2.12 (for S3 glass sample), and 3.18 (for S4 glass sample).

Using the method outlined by Archimedes in Eq. (1), the density of the synthesized S-S4 glasses was measured. The weights of the S1-S4 glasses in liquid and air are denoted by $W_{a}$ and $W_{L}$. Additionally, the density of the submerged liquid used in the current study is $\rho_{L}$≈1 g/cm^3^ for water^36^.

1 $$Density \left( {\rho ,\frac{{\text{g}}}{{{\text{cm}}^{3} }}} \right) = \frac{{W_{a} }}{{\left( {W_{a} - W_{L} } \right)}} \rho_{L}$$

Furthermore, the characterization of the S1–S4 glasses was performed using a Shimadzu-IRSpirit Fourier transform infrared (FTIR). It is used to explore the functional groups and vibration bonds for glasses within the wavenumber range from 500 to 2000 cm^−1^.

### Mechanical properties evaluations

The elastic moduli of Young (Y, GPa), bulk (K, GPa), shear (S, GPa), and longitudinal (L, GPa) as well as mechanical properties like Poisson ratio (σ), micro-hardness (H, GPa), and fractal bond conductivity (d) were estimated using the Makishima and Mackenzie theory^31,32^ based on the chemical composition and density of the fabricated lead borosilicate glasses, according to Eqs. (2–8)^37–39^.

2 $$Y = 8.36 V_{t} G_{t}$$

3 $$K = 10.0 V_{t}^{2} G_{t}$$

4 $$S = \frac{{30.0 V_{t}^{2} }}{{10.2V_{t} - 1}}G_{t}$$

5 $$L = \left[ {10 + \frac{4}{3}\left( {\frac{30}{{10.2 V_{t} - 1}}} \right)} \right]V_{t}^{2} G_{t}$$

6 $$\sigma = \frac{1}{2} - \frac{1}{{7.2V_{t} }}$$

7 $$H = \frac{{\left( {1 - 2\sigma } \right)}}{{6 \left( {1 + \sigma } \right)}}Y$$

8 $$d = \frac{{4{\text{ S}}}}{K}$$

The V_t,_ and G_t_ are the respective packing density and dissociation energy per unit volume of the utilized metal oxides.

### Monte Carlo simulation investigation

The radiation shielding parameters of the glasses under investigation were simulated and evaluated using the Monte Carlo N-particle transport code, 5th version (MCNP-5)^40^. During the simulation, the gamma-ray energy (E_γ_, MeV) was chosen to vary over a wide range, from 0.033 to 2.506 MeV, in order to encompass nearly all of the actual γ-ray energies. The ENDF/B-V.8 nuclear database, which has the interaction cross-sections needed to assess the radiation shielding capabilities of the examined glasses, was linked to the MCNP-5 code. An input file should be created with all the details required to describe the simulation components (cell, surface, material, importance, source, and cutoff cards) in order to carry out such a simulation. The mentioned input file indicates that the geometry is encircled by a 5 cm-thick lead shielding cylinder. The dry air inside the outer shielding cylinder had a density of 0.001225 g/cm^3^. Subsequently, a radioactive disk source was positioned at the center of the outer shielding cylinder (POS = 0 0 0), measuring 2 cm in diameter and 0.5 cm in thickness. A flux of γ-ray photons (PAR = 2) is released by the source along a long + Z axis (AXS = 0 0 1). The source card of the input file also included the distribution and emission probability of the radioactive source. The released photon flux was guided towards the sample using a lead collimator that measures 7 cm in height and 2 cm in diameter. The material card was modified to include the chemical compositions and densities of the materials constituting the created geometry so that the collimated photon flux could interact with the examined glasses. The fabricated glasses, shaped in cylinders, measure 3 cm in diameter and 1 cm in height. The scattered photons were then directed toward the detector through a second collimator, with a diameter of 5 cm and a height of 3 cm, after the photon flux had interacted with the electrons and atoms in the glasses. To estimate the average flux per unit glass cell and the average track length (ATL) of γ photons within the glasses under investigation, the current work uses the “F4 tally” function embedded in MCNP-5 code. By using the cutoff card, which is set up to be 10^8^, the photon-electron interaction is managed. Within the output file after the simulation runout, the simulated ATL of γ-photons was included. The mentioned output file indicates ± 1% for the relative error^41–44^. Using Eqs. (9 and 10), the linear attenuation coefficient (µ, cm^−1^) and mass attenuation coefficient (µ_m_, cm^2^/g) of the fabricated composites were calculated based on the obtained ATL of γ-photons.‬‬

9 $$\mu ({\text{cm}}^{ - 1} ) = \frac{1}{x}ln\left( {\frac{{I_{o} }}{{I_{t} }}} \right)$$

10 $$\mu_{m} \left( {\frac{{{\text{cm}}^{2} }}{{\text{g}}}} \right) = \frac{{\mu \left( {{\text{cm}}^{ - 1} } \right)}}{{\rho \left( {\frac{{\text{g}}}{{{\text{cm}}^{3} }}} \right)}}$$

where I_o_ and I_t_ values refer to the photon flux before and after interaction with the fabricated glasses.

The half-value thickness (Δ_0.5_, cm) describes the thickness of the material required to diminish the photon flux by 50%. It is inversely varied with the µ values according to Eq. (11).

11 $$HVL = \frac{ln \left( 2 \right) }{{\mu \left( { {\text{cm}}^{ - 1} } \right)}}$$

Additionally, the transmission factor (TF, %) for the fabricated S1-S4 glasses was estimated according to Eq. 12, where the $\frac{{I_{o} }}{{I_{t} }}$ represents the ratio of transmitted photons. On the contrast, the RPE (%) describes the amount of photons absorbed within the fabricated glasses thickness. It is calculated based on Eq. 13.

12 $$TF\left( \% \right) = \frac{{I_{o} }}{{I_{t} }} \times 100$$

13 $$RPE\left( \% \right) = \frac{{I_{a} }}{{I_{t} }} \times 100 = \frac{{(I_{o} - I_{t} )}}{{I_{t} }} \times 100$$

In addition to being evaluated through Monte Carlo simulation, the µ_m_ was theoretically calculated with XCOM software. The (µ_m_)_glass_ can be theoretically computed using Eq. (14)^45^.

14 $$\left( {\mu_{m} } \right)_{glass} = \mathop \sum \limits_{i} w_{i} \left( {\mu_{m} } \right)_{i}$$

ρ, ω_i_, and ($\mu_{m}$)_i_ describe the density of fabricated glasses, the weight fraction of i^th^ element within the glass sample, and µ_m_ for the i^th^ constituent element, respectively.

## Results and discussion

**Figure 2:**
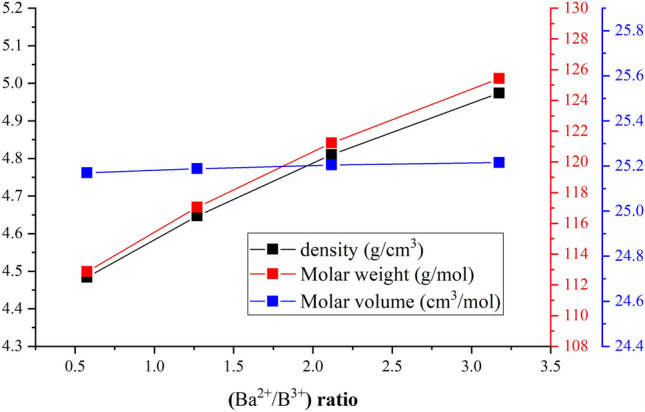
Impact of Ba^2+^/B^3+^ ratio on theoretical density, molar weight, and molar volume of the fabricated glasses.

The substitution of B_2_O_3_ by the BaO compound increases the Ba^2+^ ions while it decreases the B^3+^ ions in the boro-silicated glasses. This affects the color of the synthesized boro-silicate glasses. The transparent color is transferred to a light yellow and then to a dark yellow color by increasing the Ba^2+^/B^3+^ ratio between 0.58 (S1 glass sample) and 3.18 (S-4 glass sample), as illustrated in Fig. 1. The ratio of Ba/B plays an important role in controlling the density, molar weight, and molar volume of the fabricated glasses, as illustrated in Table 1 and Fig. 2. The increase in BaO concentration increases the Ba^2+^ ions, which in turn increases the Ba/B ratio due to the substitution of B^3+^ by Ba^2+^ ions. The increase in Ba^2+^/B^3+^ ratio is associated with an increase in the molar weight of the fabricated glasses, where the molar weight increases from 112.86 to 125.42 mol/g, when rising the Ba^2+^/B^3+^ ratio between 0.58 and 3.18, respectively. Moreover, the experimental measurements of the fabricated glass density show an increase from 4.48 to 4.97 g/cm^3^, when rising the Ba/B ratio between 0.58 and 3.18, respectively. The increase in the fabricated glasses density is attributed to the partial replacement of Ba (ρ = 3.34 g/cm^3^) for the B (ρ = 2.46 g/cm^3^) ions. The increase in the density and molar weight of the developed glasses is found to be accompanied by a negligible increase in the molar volume of the fabricated glasses, which varies from 25.17 to 25.21 cm^3^/mol. The aforementioned negligible increase in the V_m_ values is attributed to the close comparable V_m_ values for both B_2_O_3_ and BaO compounds, where the (V_m_)_B2O3_ = 28.30 cm^3^/mol and (V_m_)_BaO_ = 26.805 cm^3^/mol.

**Table 1:** Chemical composition of the prepared glass samples.

Sample	Al_2_O_3_	PbO	SiO_2_	B_2_O_3_	BaO	Theoretical Density (g/cm^3^)
S1	5	25	10	55	5	4.484
S2	5	25	10	50	10	4.647
S3	5	25	10	45	15	4.810
S4	5	25	10	40	20	4.973

**Figure 3:**
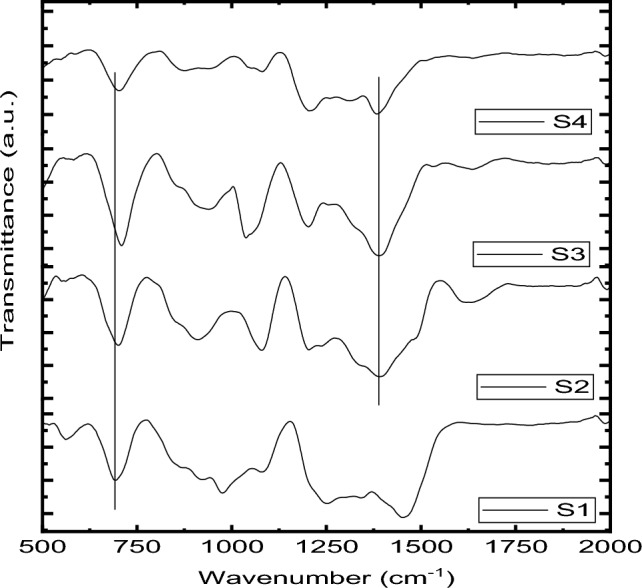
FTIR results for Al_2_O_3_-PbO-B_2_O_3_-SiO_2_-BaO glasses.

Fourier transform infrared (FTIR) is an instrument used to analyze functional groups and provide information about chemical bonding and molecular structure for various materials. Table 2 and Fig. 3 illustrate the functional groups for the glass system. It can be noted in four bands. A small band is located at 550 and 560 cm^−1^, which is related to the O–Si–O bending vibration mode^35^, while the band appearance at 690 and 703 cm^−1^ can be assigned to bending vibration B–O–B^46^. The large band centered at 906 to 971 cm^−1^ and 1039 to 1076 cm^−1^ can correspond to the B–O stretching of the tetrahedral BO_4_ unit^48^ The last band at high wavenumbers from 1388 to 1453 cm^−1^ and 1202 and 1247 cm^−1^ is associated with B–O stretching of trigonal BO_3_^47^. On the other hand, it can be noted that the change in band position for the BO_3_ band with adding a further amount of BaO is due to transforming BO_3_ to BO_4_ and forming nonbridging oxygen. Here, it can be concluded that the BaO plays a modifier in the glass system.

**Table 2:** FTIR assignments for Al_2_O_3_–PbO–B_2_O_3_–SiO_2_–BaO glasses.

Vibration bond and functional groups (cm^−1^)	Glass samples			
	S1	S2	S3	S4
O–Si–O bending vibration mode	550	550	560	560
Bending vibration B–O–B	690	701	703	701
B–O stretching of tetrahedral BO_4_ unit	1076, 971	1076, 906	1039, 947	1076, 938
B–O stretching of trigonal BO_3_	1453, 1247	1388, 1202	1388, 1202	1388, 1202

Table 3 depicts the variation in the mechanical properties of the fabricated glasses with the increasing of Ba^2+^/B^3+^ ratio. Rising the Ba^2+^/B^3+^ ratio between 0.58 (for S1) and 3.18 (for S4) is found to reduce the total dissociation energy G_t_ from 60.87 to 54.37 kcal/cm^3^ and the packing density V_t_ from 0.57 to 0.50 m^3^/mol. The reduction in the G_t_ and V_t_ values are attributed to the packing factor V_i_ and dissociation energy (G_i_) for both B_2_O_3_ and BaO compounds, where the B_2_O_3_ compound has G_i_ = 82.8 kcal/cm^3^ and V_i_ = 20.8 m^3^/mol while for BaO compound G_i_ = 39.5 kcal/cm^3^ and V_i_ = 9 m^3^/mol^48^. The replacement of B_2_O_3_ (high G_t_ and V_i_ values) with BaO (low G_i_ and V_i_ values) is the main reason for the reduction in both G_t_ and V_t_ of the fabricated glasses. The reduction in the G_t_ and V_t_ for the fabricated glasses reflects on the mechanical moduli. The increase of substitution ratio Ba/B between 0.58 and 3.18 decreases the moduli from 69.35 to 54.21 GPa (for Y modulus), from 47.41 to 32.43 GPa (for K modulus), from 27.60 to 22.19 GPa (for S modulus), and from 84.22 to 62.02 GPa (for L modulus). Additionally, the Poisson ratio is slightly reduced from 0.26 to 0.22, associated with a similar reduction in the micro-hardness of the fabricated glasses from 4.49 to 4.12 GPa. After that, the increase in the fractal bond conductivity from 2.33 to 2.74 confirms a transformation of the glassy structure to a 3D network.

**Table 3:** The mechanical properties for the fabricated BaO doped lead borosilicate glasses.

	Mechanical properties								
	G_t_ (kcal/cm^3^ )	V_t_	Y (GPa)	K (GPa)	S (GPa)	σ	H (GPa)	L (GPa)	fractal bond conductivity (d)
S1	60.87	0.57	69.35	47.41	27.60	0.26	4.49	84.22	2.33
S2	58.70	0.55	64.09	41.98	25.73	0.25	4.36	76.28	2.45
S3	56.54	0.52	59.04	36.99	23.92	0.23	4.24	68.88	2.59
S4	54.37	0.50	54.21	32.43	22.19	0.22	4.12	62.02	2.74

**Figure 4:**
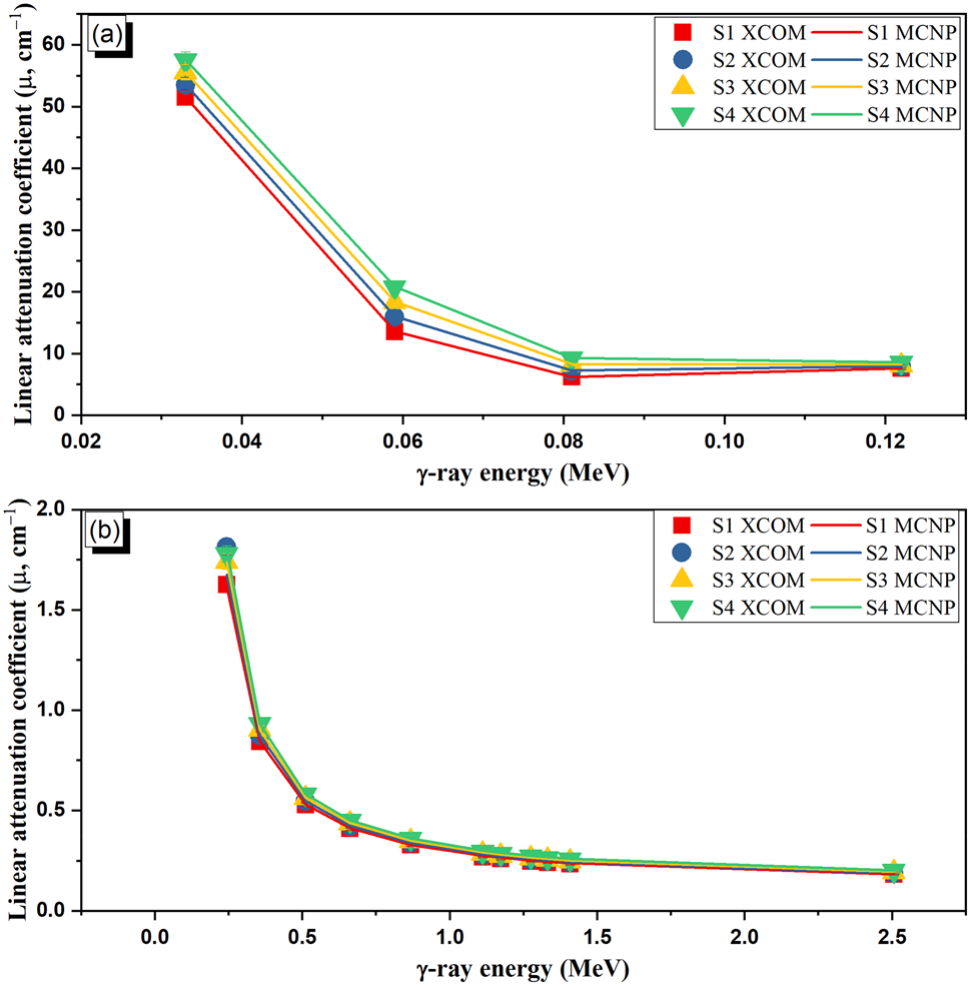
Variation of linear attenuation coefficient (µ, cm^−1^) versus the photon energy at γ-ray interaction modes: (**a**) photoelectric interaction and (**b**) Compton scattering interactions.

The radiation shielding properties of the fabricated glasses were investigated utilizing the Monte Carlo simulation (MCNP-5) code and the XCOM theoretical program, as illustrated in Fig. 4 (a and b). The gamma-ray shielding evaluations show a dependence of the shielding ability on some parameters related to the γ-ray source and the examined materials. Regarding the γ-ray source, the source energy (E_γ_, MeV) greatly affects the µ values of the fabricated glasses, where greeting the E_γ_ is associated with a reduction in the µ values. The reduction in the µ values is a result of the reduction in the interaction cross-section of γ-photons, where the cross-section varied with $E_{\gamma }^{ - 3.5}$ and $E_{\gamma }^{ - 1}$ for photoelectric (PE) and Compton scattering (CS) interactions^49^. Figure 4**-a** shows that the high µ values are 51.51 cm^−1^, 53.52 cm^−1^, 55.52 cm^−1^, and 57.52 cm^−1^ for samples S1, S2, S3, and S4, respectively, at 0.033 MeV. Then, the µ values obtained at 0.033 MeV were reduced by approximately 85.3% for all samples when the E_γ_ values are increased to 0.122 MeV. This high reduction is attributed to the PE cross-section. After that, when increasing the E_γ_ values above 0.122 MeV, the µ decreased by 88.8%, 89.6%, 88.8%, and 88.7%, respectively, S1, S2, S3, and S4. In particular, the reduction in the µ values, for E_γ_ interval between 0.244 and 2.506 MeV, is due to the CS interaction^50^.

Based on the measured µ, the µ_m_ values were calculated for the fabricated glasses, the results are grouped in Table 4. The µ_m_ values reduced in the interval between 11.488–0.041 cm^2^/g for sample S1, 11.516–0.040 cm^2^/g for sample S2, 11.542–0.040 cm^2^/g for sample S3, and 11.567–0.040 cm^2^/g for sample S4, all when rising the E_γ_ values from 0.033 to 2.506 MeV. Moreover, Table 4 shows an agreement between the simulated MCNP and XCOM calculated µ_m_ values with a difference of less than ± 2% in average.

**Table 4:** Comparison between the simulated MCNP-5 and XCOM theoretical calculated µ_m_ values for the fabricated glasses.

Energy (MeV)	Mass attenuation coefficient (µ_m_, cm^2^/g)											
	S1			S2			S3			S4		
	MCNP-5	XCOM	Diff (%)	MCNP-5	XCOM	Diff (%)	MCNP-5	XCOM	Diff (%)	MCNP-5	XCOM	Diff (%)
0.033	11.488	11.500	− 0.105	11.516	11.530	− 0.119	11.542	11.560	− 0.153	11.567	11.590	− 0.202
0.059	3.023	3.041	− 0.588	3.433	3.451	− 0.517	3.815	3.832	− 0.447	4.171	4.187	− 0.378
0.081	1.384	1.390	− 0.460	1.538	1.562	− 1.563	1.700	1.721	− 1.265	1.849	1.870	− 1.123
0.122	1.694	1.694	0.023	1.709	1.707	0.115	1.688	1.719	− 1.867	1.708	1.730	− 1.261
0.244	0.363	0.360	0.666	0.390	0.361	7.651	0.361	0.361	0.151	0.357	0.361	− 1.097
0.356	0.188	0.188	− 0.167	0.188	0.188	0.146	0.188	0.187	0.080	0.187	0.187	0.042
0.511	0.118	0.118	− 0.490	0.117	0.118	− 0.454	0.117	0.118	− 0.471	0.117	0.117	− 0.465
0.662	0.091	0.092	− 0.437	0.091	0.091	− 0.442	0.090	0.091	− 0.444	0.090	0.090	− 0.449
0.867	0.073	0.073	− 0.430	0.073	0.073	− 0.434	0.072	0.073	− 0.440	0.072	0.072	− 0.444
1.112	0.060	0.061	− 2.226	0.060	0.061	− 2.265	0.059	0.061	− 2.323	0.059	0.060	− 2.361
1.173	0.058	0.059	− 2.070	0.058	0.059	− 2.122	0.057	0.059	− 2.179	0.057	0.058	− 2.213
1.275	0.055	0.056	− 1.818	0.055	0.056	− 1.865	0.055	0.056	− 1.921	0.054	0.055	− 1.955
1.332	0.054	0.055	− 1.763	0.053	0.054	− 1.806	0.053	0.054	− 1.841	0.053	0.054	− 1.894
1.408	0.052	0.053	− 1.663	0.052	0.053	− 1.715	0.052	0.052	− 1.741	0.051	0.052	− 1.788
2.506	0.041	0.041	− 0.881	0.040	0.041	− 0.887	0.040	0.041	− 0.899	0.040	0.041	− 0.923

**Figure 5:**
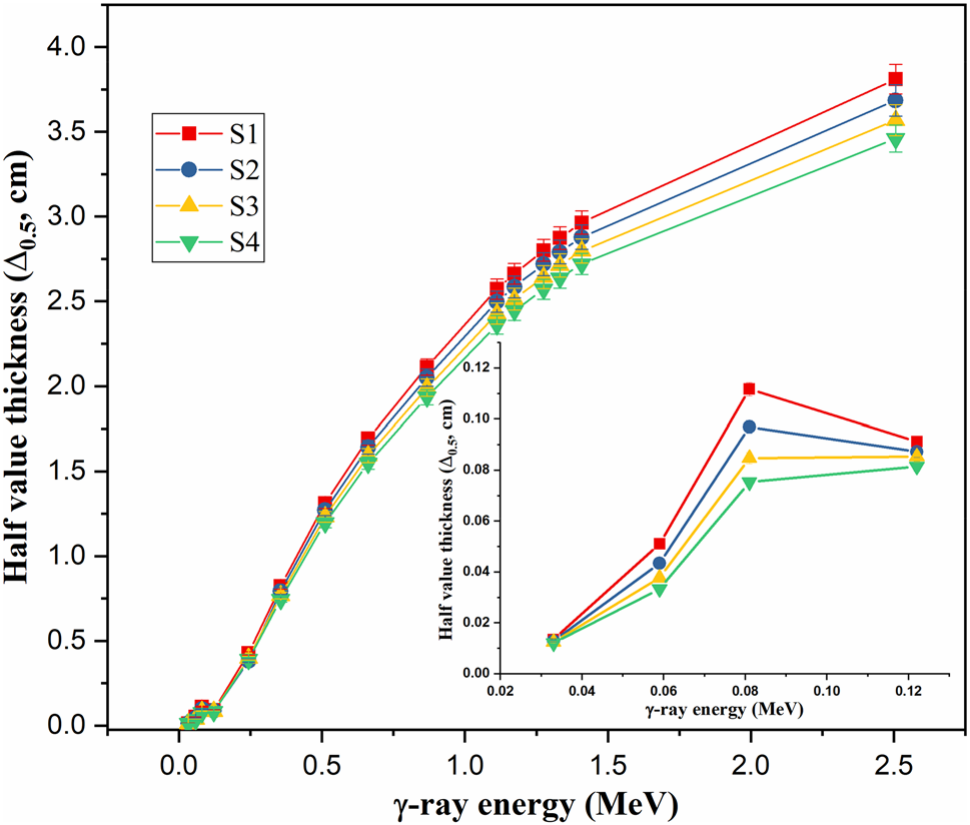
Variation of the half value thickness (Δ_0.5_, cm) against the γ-photon energy.

The reduction in µ values due to the E_γ_ increase is followed by an increase in the Δ_0.5_ values, as illustrated in Fig. 5. The Δ_0.5_ values increased under the effect of PE and CS interactions between 0.0–3.81 cm for sample S1, 0.01–3.69 cm for sample S2, 0.01–3.57 cm for sample S3, and 0.01–3.46 cm for sample S4, with rising E_γ_ between 0.033–2.506 MeV. As illustrated earlier, the increase in the E_γ_ values reduces the PE and CS cross-sections, leading to a reduction in the photon-electron interactions^51^. Therefore, the transmitted photons I_t_ increased while the absorbed photons I_a_ increased, resulting in a reduction in the µ values and an increase in the Δ_0.5_ values of the fabricated glasses, where µ = 0.693/Δ_0.5_.

**Figure 6:**
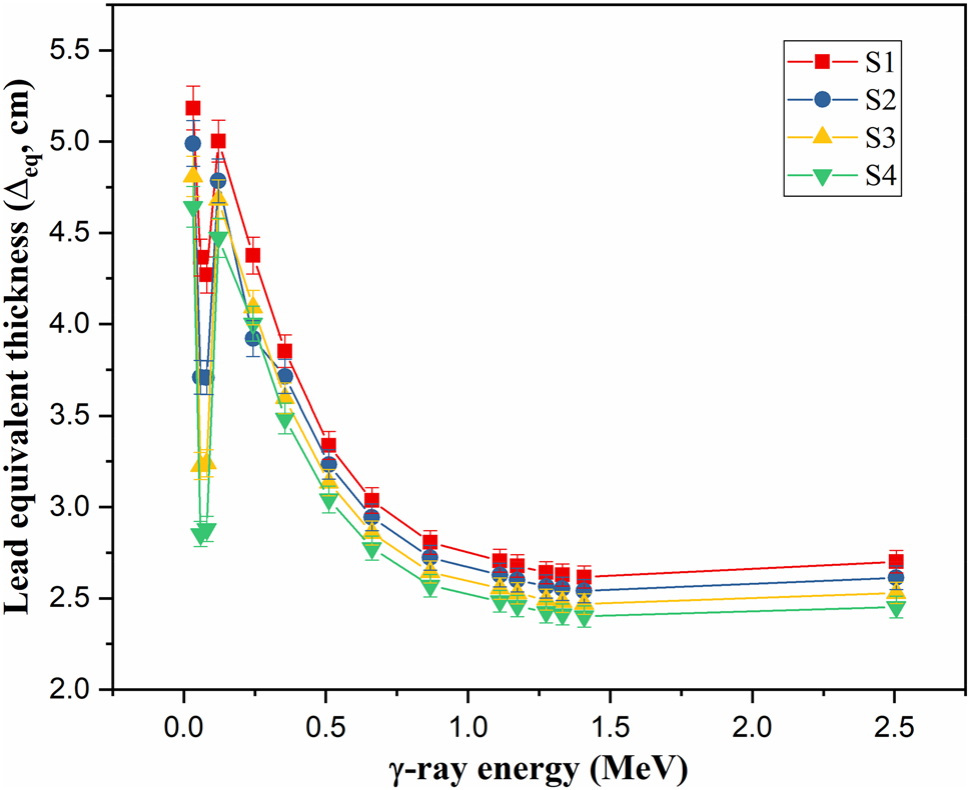
Variation of the lead equivalent thickness (Δ_eq_, cm) against the γ-photon energy.

The mode of variation in the µ values for the fabricated samples affects their Δ_eq_ values, where the increase in E_γ_ values decreases the Δ_eq_ of the fabricated glasses, as presented in Fig. 6. The reduction in the Δ_eq_ values is attributed to the comparable reduction in both µ values for Pb and fabricated glasses. While increasing the E_γ_ values in the PE interval, the µ values of all tested samples were reduced by approximately 85.3%. Simultaneously, the increase in the E_γ_ values in the same PE energy interval (0.033 MeV ≤ E ≤ 0.122 MeV) decreases the µ values for lead by 85.8%. The comparable reduction in the µ values for both fabricated glasses and Pb is the main reason behind the exponential reduction in the Δ_eq_ values. Additionally, due to the K-absorption of Pb, the Δ_eq_ values increased around 0.081 MeV because of the high µ values of lead at this energy. In the PE interval, the Δ_eq_ values were reduced by 15.58%, 21.41%, 14.93%, and 13.78% for samples S1, S2, S3, and S4, respectively. Furthermore, the increase in E_γ_ values above 0.122 MeV (i.e., CS interval) leads to a moderate reduction in the Δ_eq_ values. This reduction was achieved due to the moderate reduction in the µ values for both fabricated samples and Pb, obtained by rising the E_γ_ between 0.244 and 2.506 MeV, where the µ value of lead was reduced by 93.1%, while the µ values were reduced by 88.8%, 89.6%, 88.8%, and 88.7% for samples S1, S2, S3, and S4, respectively. In the CS interaction interval, while rising the E_γ_ values between 0.244 and 2.506 MeV, the Δ_eq_ for S1, S2, S3, and S4 was reduced by 38.28%, 33.37%, 38.16%, and 38.72%, respectively.

**Figure 7:**
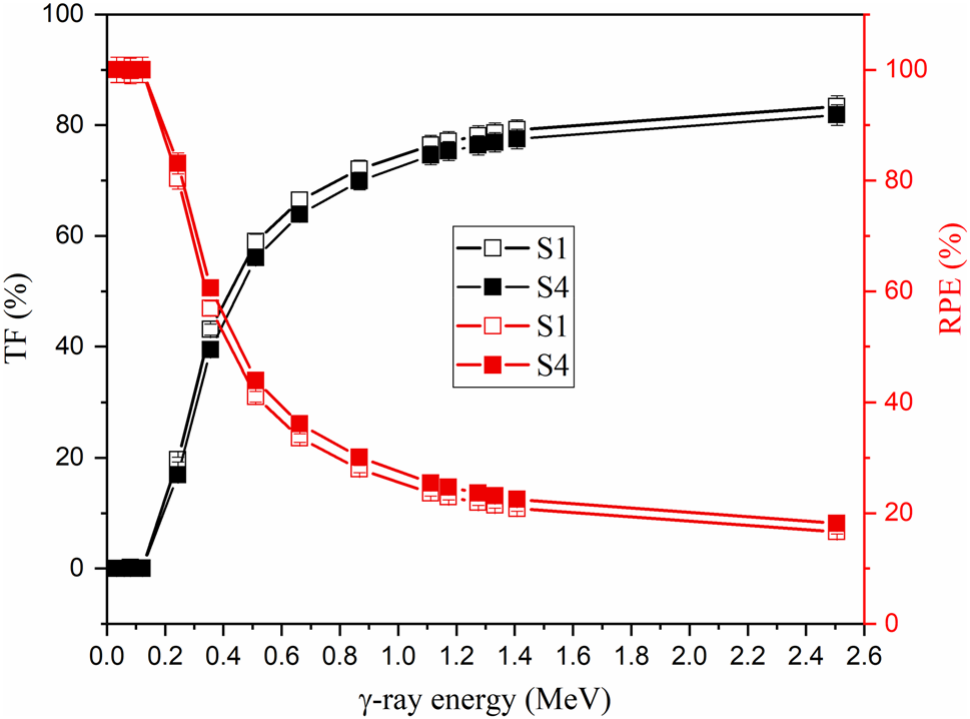
Impact of the applied γ-photon energy on TF and RPE values, for the fabricated glasses S1 and S4.

The increase in E_γ_ values is accompanied by an increase in the I_t_ photons and a reduction in the I_a_ photons. Since the I_t_/I_o_ ratio determines the TF value and the I_a_/I_o_ determines the RPE values, the TF value increased while the RPE values decreased with rising the E_γ_ value, as presented in Fig. 7. Due to the PE interaction behavior at low energy, the photon energy was transferred to one electron, and the photon disappeared in the medium, leading to a reduction in the I_t_ photons and TF values^52^. The TF values in the interval between 0.033 and 0.122 MeV are less than 1% for a 1 cm thickness of the fabricated samples S1 and S4. In contrast, the I_a_ photon number increases and reaches its maximum, leading to an increase in the RPE values, where the RPE values are close to 100% for all samples. Increasing the E_γ_ values between 0.244 and 2.506 MeV is associated with a high increase in I_t_ photons and a reduction in the I_a_ values due to the CS behavior. Therefore, the TF was highly increased, while the RPE values were reduced with rising E_γ_ values. For example, increasing the E_γ_ values between 0.244 and 2.506 MeV increases the TF values of a 1 cm thickness of the fabricated glasses between 19.68–83.37% for sample S1 and 16.91–81.84% for sample S4. On the other hand, the RPE values were reduced by a factor ranging between 80.32–16.63% for sample S1 and 83.09–18.16% for sample S2, when rising the E_γ_ between 0.244 and 2.506 MeV.

**Figure 8:**
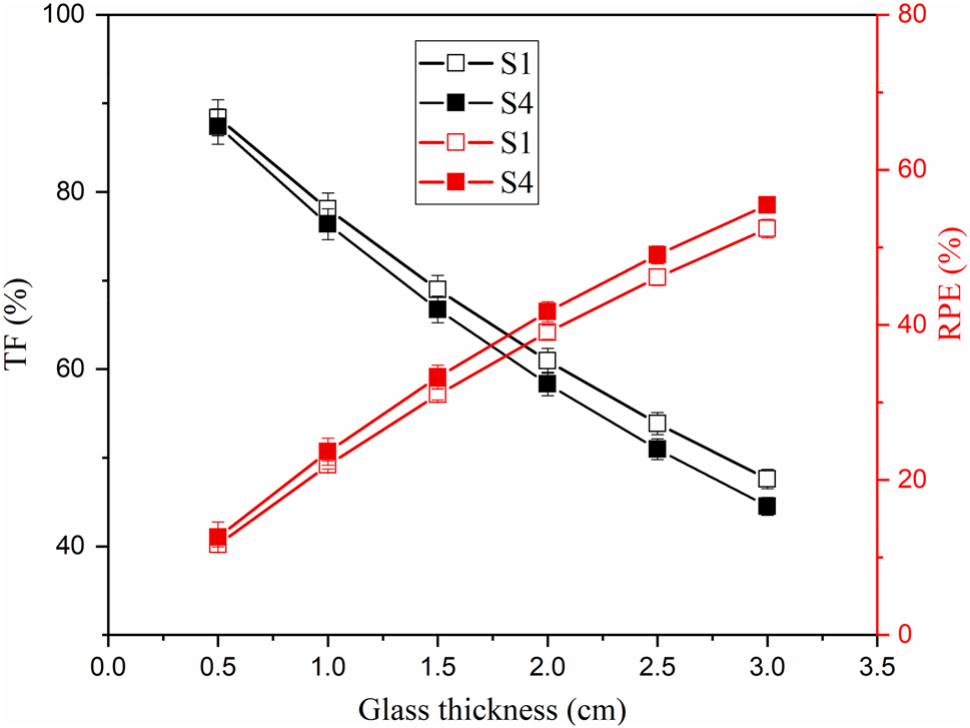
Impact of the glass thickness on TF and RPE values for the fabricated glasses, at 1.275 MeV.

The glass thickness also greatly affects the values of I_t_ and I_a_ which then affect the TF and RPE values. Rising the fabricated glass thickness reduces the TF, while increasing the RPE of the fabricated glasses, as shown in Fig. 8. In fact, the increase in glass thickness increases the pass length of γ-photons, which leads to an increase in the interaction probability between photons and surrounding electrons^53^. Therefore, the I_a_ photons increased and the I_t_ photons decreased, leading to an increase in the RPE and a reduction in the TF values. For example, increasing the glass thickness from 0.5 to 3 cm increases the RPE values at E_γ_ of 1.275 MeV between 11.64–52.41% for sample S1 and 12.61–55.47% for sample S4. On the other hand, for the same thickness variation, the TF values decreased by 46.14% and 49.04% for samples S1 and S4, respectively.

**Figure 9:**
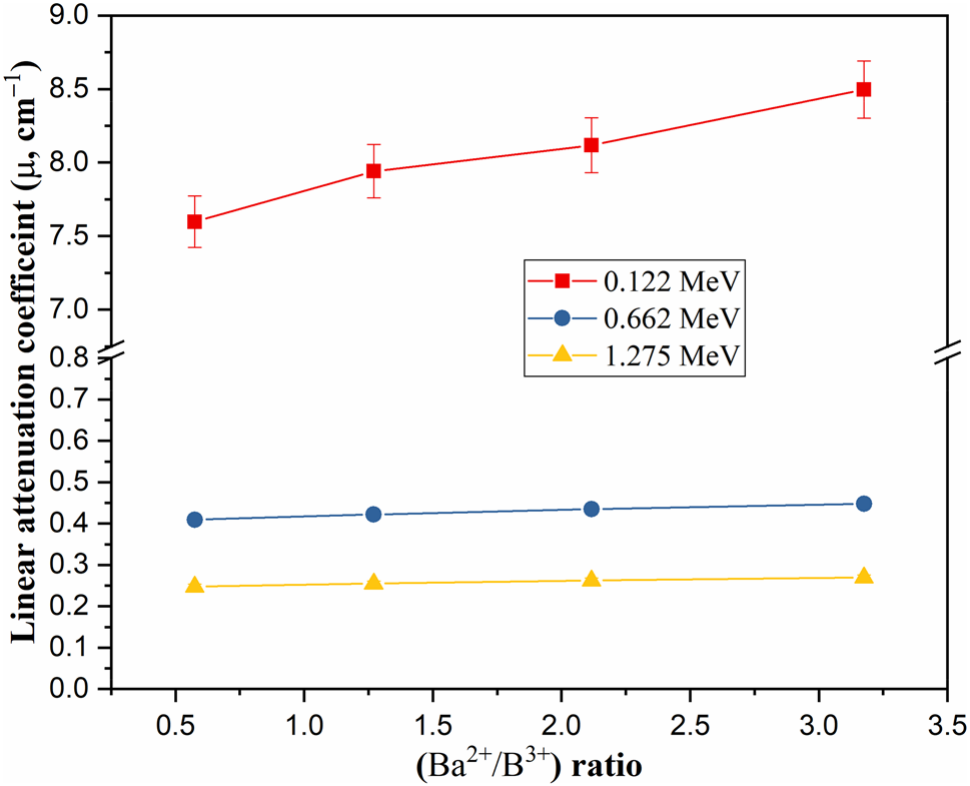
Impact of the Ba^2+^/B^3+^ ratio on the linear attenuation coefficient (µ, cm^−1^) of the fabricated at E_γ_ values of 0.122, 0.662, and 1.275 MeV.

**Figure 10:**
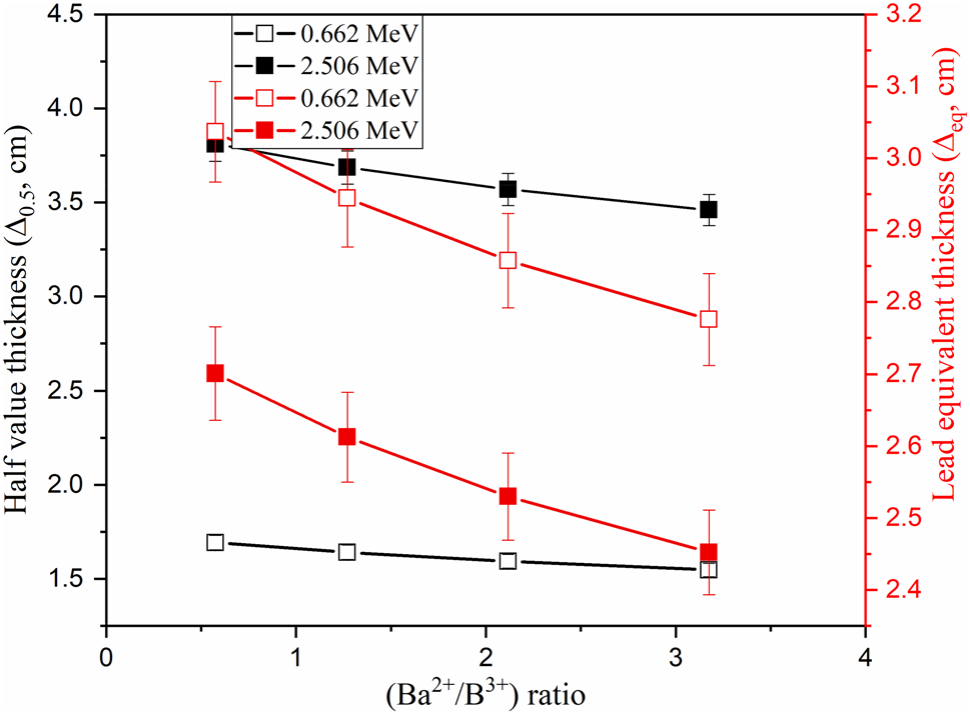
Impact of the Ba^2+^/B^3+^ ratio on the half value thickness (Δ_0.5_, cm) and equivalent thickness of lead (Δ_eq_, cm), at E_γ_ of 0.662 and 2.506 MeV.

**Figure 11:**
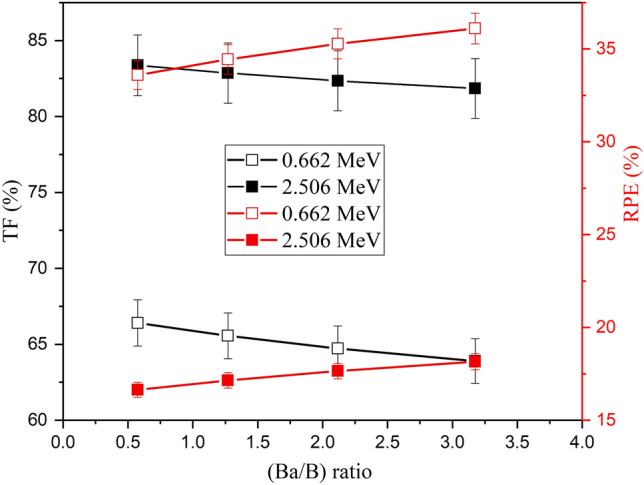
Impact of the Ba^2+^/B^3+^ ratio on TF (%) and RPE (%) values, at E_γ_ of 0.662 and 2.506 MeV.

Increasing the substitution of B_2_O_3_ by BaO compounds increases the Ba/B ratio within the fabricated glasses, which affects the glass density and its molar weight, as presented earlier. The impact of the Ba^2+^/B^3+^ ratio on the µ values is illustrated in Fig. 9, where increasing the Ba/B ratio is found to slightly increase the µ values. Increasing the Ba^2+^/B^3+^ ratio also increases the electron density and Z_eff_ of the fabricated glasses. Since the interaction cross-section proportion to Z_eff_ increased, the µ values increased as the Ba^2+^/B^3+^ increased. Figure 9 shows that the increase in Ba/B ratio from 0.58 to 3.18 is associated with an increase in the µ values by 11.8%, 9.4%, and 8.94%, respectively, at E_γ_ of 0.122, 0.662, and 1.275 MeV. The impacts of Ba^2+^/B^3+^ on the Δ_0.5_ and Δ_eq_ values are opposite to those reported for the µ values. Figure 10 shows a reduction in the Δ_0.5_ values from 1.69 to 1.55 cm (at E_γ_ of 0.662 MeV) and from 3.81 to 3.46 cm (at E_γ_ of 2.506 MeV), when rising the Ba/B ratio between 0.58 and 3.18, respectively. The reduction in the Δ_0.5_ values is attributed to the reverse proportionality of µ and Δ_0.5_ values. Also, the Δ_eq_ values were reduced, while rising the Ba^2+^/B^3+^ ratio, where the Δ_eq_ values reduced from 3.04 to 2.78 cm at E_γ_ of 0.662 MeV and from 2.70 to 2.45 cm at E_γ_ of 2.506 MeV. The increase in the Ba^2+^/B^3+^ ratio increases the Ba^2+^ ions within the fabricated glasses, which increases the resistance of the material to the transposed photons. Therefore, the number of photon-electron interactions increased, I_t_ decreased, and I_a_ and µ values increased. The increase in µ values of the fabricated glasses compared to the µ values of lead is the main reason for the reduction of Δ_eq_. Furthermore, the increase in I_a_ photons and the decrease in I_t_ photons affect the values of TF and RPE, as illustrated in Fig. 11. The increase in the Ba/B ratio from 0.58 to 3.18 decreases the TF values from 66.41% to 63.90% for E_γ_ of 0.662 MeV and from 83.37% to 81.84% for E_γ_ of 2.506 MeV. In comparison, the RPE values increase from 33.59% to 36.10% for E_γ_ of 0.662 MeV and from 16.63% to 18.16% for E_γ_ of 2.506 MeV.

**Figure 12:**
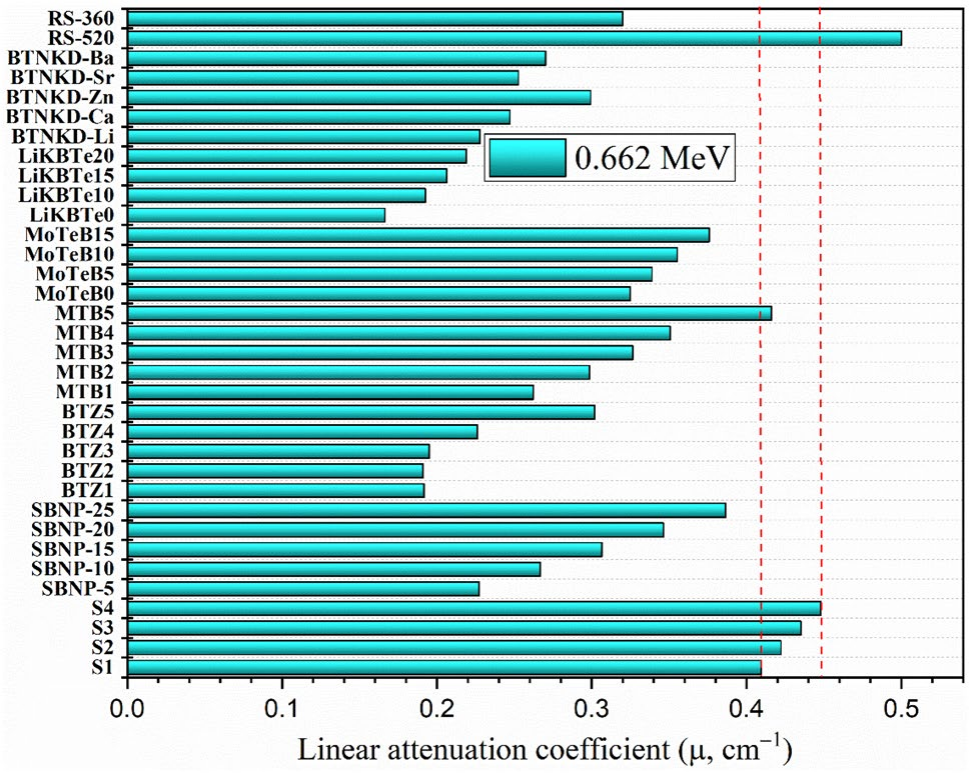
Comparison between the linear attenuation coefficient of the developed glasses and some commercial and recently reported borate-based glasses.

To verify the capacity of the developed S1-S4 glasses to block the intermediate gamma rays energies, the µ values for the developed S1-S4 glasses at 0.662 MeV were compared to those reported commercial glasses (RS-520 and RS-360)^54^ and borate-based glasses for gamma ray shielding applications reported previously in various publications^55–60^, as illustrated in Fig. 12.

The developed glasses µ values at 0.662 MeV are 0.409 cm^−1^, 0.422 cm^−1^, 0.435 cm^−1^, and 0.448 cm^−1^, for glass samples S1, S2, S3, and S4, respectively. The aforementioned µ values for the developed glasses in the current study are lower than that reported for the commercial shielding glass RS-520 (µ = 0.50 cm^−1^) at 0.662 MeV. The high µ value for the RS-520 glass sample is attributed to its high content of PbO, which reaches 71 wt.% of its compositions^54^. Then, the fabricated glasses S1-S4 have µ values higher than that reported for commercial glass RS-360 (µ = 0.32 cm^−1^), which contains 45 wt.% of PbO in its composition^54^. Additionally, the developed S1-S4 glass samples have µ values higher than that reported for previously reported glasses SBNP-5 (µ = 0.227 cm^−1^), SBNP-10 (µ = 0.267 cm^−1^), SBNP-15 (µ = 0.307 cm^−1^), SBNP-20 (µ = 0.346 cm^−1^), SBNP-25 (µ = 0.386 cm^−1^), BTZ1 (µ = 0.192 cm^−1^), BTZ2 (µ = 0.191 cm^−1^), BTZ3 (µ = 0.195 cm^−1^), BTZ4 (µ = 0.226 cm^−1^), BTZ5 (µ = 0.302 cm^−1^), MTB1 (µ = 0.262 cm^−1^), MTB2 (µ = 0.299 cm^−1^), MTB3 (µ = 0.327 cm^−1^), MTB4 (µ = 0.350 cm^−1^), MoTeB0 (µ = 0.325 cm^−1^), MoTeB5 (µ = 0.339 cm^−1^), MoTeB10 (µ = 0.355 cm^−1^), MoTeB15 (µ = 0.376 cm^−1^), LiKBTe0 (µ = 0.166 cm^−1^), LiKBTe10 (µ = 0.192 cm^−1^), LiKBTe15 (µ = 0.206 cm^−1^), LiKBTe20 (µ = 0.219 cm^−1^), BTNKD-Li (µ = 0.228 cm^−1^), BTNKD-Ca (µ = 0.247 cm^−1^), BTNKD-Zn (µ = 0.299 cm^−1^), BTNKD-Sr (µ = 0.253 cm^−1^), and BTNKD-Ba (µ = 0.270 cm^−1^)^55–60^. The fabricated glass S1 with the lowest µ values in the current study is close to the µ values for the previously reported glass MTB5 (µ = 0.416 cm^−1^). The aforementioned MTB5 sample has high concentrations of dense metal oxides TeO_2_ and BaO, where their ratios reach 70 mol% and 20 mol%, respectively. The comparison shows high shielding capacity for the developed glasses compared to the commercial-based PbO compounds and those glasses reported recently for gamma ray shielding applications. Therefore, the fabricated glasses are suitable candidates for mid-energy gamma-ray shielding applications.

## Conclusion

The current study concludes with the efficiency of new BaO-doped lead borosilicate glasses adopted for gamma-ray shielding applications. The effects of partially replacing B^3+^ with Ba^2+^ ions on the developed glasses' mechanical, gamma-ray attenuation, and physical characteristics were assessed. The color of the fabricated glasses was turned to dark yellow with increasing the Ba^2+^/B^3+^ substitution ratio. Additionally, the density of the fabricated glasses enhanced by 11% from 4.48 to 4.97 g/cm^3^, increasing the Ba^2+^/B^3+^ substitution ratio from 0.58 to 3.18, respectively. The replacement of B^3+^ ions by Ba^2+^ ions reduces the mechanical moduli of the developed glasses, where they reduced by 21.84%, 31.61%, 19.61%, and 26.36% for Young, bulk, shear, and longitudinal moduli when the Ba^2+^/B^3+^ substitution ratio increased between 0.58 and 3.18. Also, the micro-hardness of the fabricated samples decreased by 8.12% (between 4.49 and 4.12 GPa). The reduction observed in the mechanical properties is attributed to the reduction in the packing density and dissociation energy due to the substitution of B by Ba ions. Additionally, the Monte Carlo simulation proves that the linear attenuation coefficient of the fabricated glasses was enhanced by 48.23%, 11.83%, 9.40%, and 10.13%, rising the Ba/B substitution ratio between 0.58 and 3.18, respectively. The enhancement in the linear attenuation coefficient reduces the half-value thickness and the equivalent thickness for lead. Compared to the shielding capacity of some commercial glasses and borate-based glasses, the developed glasses S1-S4 have suitable radiation shielding properties to be used in nuclear medicine applications at hospitals.

## Data Availability

All data generated or analyzed during this study are included in this published article.
